# Sperm quality after density gradient centrifugation with three commercially available media: a controlled trial

**DOI:** 10.1186/1477-7827-12-121

**Published:** 2014-12-02

**Authors:** Helena Malvezzi, Rakesh Sharma, Ashok Agarwal, Adel M Abuzenadah, Muhammad Abu-Elmagd

**Affiliations:** Center for Reproductive Medicine, Glickman Urological & Kidney Institute, Cleveland Clinic, Cleveland, OH USA; Centre of Excellence in Genomic Medicine Research, King Abdulaziz University, Jeddah, Saudi Arabia; KACST Technology Innovation Center in Personalized Medicine at King AbdulAziz University, Jeddah, Saudi Arabia; Zoology Department, Faculty of Science, Minia University, Minia, Egypt

**Keywords:** Sperm motility, Sperm processing, Reproductive potential, Density gradient centrifugation, DNA fragmentation

## Abstract

**Background:**

Density gradient is the preferred technique for sperm processing for ART. However, no study has examined sperm quality using different processing media simultaneously and under identical conditions. Therefore, we evaluated semen quality following sperm preparation by three commonly used commercially available density gradient media in a well-designed controlled trial.

**Methods:**

We obtained semen samples from 20 healthy volunteers. Percent motility, total motile sperm (TMS), % recovery and DNA damage were assessed before and after separation in three different sperm density gradient media-PureCeption, ISolate and SpermGrad-125.

**Results:**

Percent motility was higher in the ISolate (81.4% ± 6.6%) and SpermGrad-125 samples (85.7% ± 8.0%) (P < 0.0001) than in the PureCeption samples (62.5% ± 13.2%) (P = 0.07). TMS was higher in the PureCeption(TM) and ISolate samples (14.2% ± 15.9% and 15.8% ± 18.2%) than in those prepared with SpermGrad-125 (10.6% ± 19.7%) (P < 0.0001). Percent recovery was significantly higher in the PureCeption(TM) and ISolate samples (45.3% and 48.9%) than in the SpermGrad-125(TM) samples (30.8%) (P < 0.01). DNA fragmentation was comparable across the three gradients (PureCeption = 8.8% ± 4.7%; ISolate = 7.2 ± 5.2% and SpermGrad-125 = 11.2% ± 7.4%).

**Conclusions:**

Three different density gradient processing media PureCeption, ISolate, and SpermGrad-125 were examined for their effects on sperm quality. Sperm processed by ISolate and Sperm Grad 125 had better motility and TMS after processing. The extent of DNA damage was comparable in all three gradients.

## Background

Before sperm can be used in assisted reproductive techniques (e.g. intrauterine insemination or classic in vitro fertilization alone or combined with intracytoplasmic sperm injection), it must first be processed. The goal of processing is to select highly motile, morphologically normal spermatozoa with minimal DNA damage [1–3]. At the same time, cellular debris must be removed, including round cells, epithelial cells, white blood cells as well as immature or morphologically abnormal spermatozoa that are immotile or have poor motility. Migration based protocols include the swim up or the swim down techniques. In the swim up technique, the highly motile sperm migrate out into the clear culture medium [4, 5]. In the swim-down technique the motile sperm migrate down the discontinuous gradient commonly prepared using bovine serum albumin. Spermatozoa are layered on the top of the albumin gradient. Although this is a relatively inexpensive technique the sperm recovery is very poor.

Sperm preparation by density gradient centrifugation separates sperm cells based on their density. Morphologically normal and abnormal spermatozoa have different densities. A mature morphologically normal spermatozoon has a slightly higher density of 1.10 g/mL whereas an immature and morphologically abnormal spermatozoon has a lower density between 1.06 and 1.09 g/mL. At end of centrifugation, each spermatozoon is situated at the gradient level that matches its density. As a result, the resulting interphases between seminal plasma and the 40% upper layer, containing the leukocytes, cell debris and 40% and 80% containing morphologically abnormal sperm with poor motility are discarded. The highly motile, morphologically normal, viable spermatozoa form a pellet at the bottom of the tube [4, 5]. Although sperm preparation can be accomplished using a number of techniques such as simple wash, swim-up and density gradient [5, 6], research shows that the latter consistently produces samples of the highest quality required for intrauterine insemination and for in vitro fertilization (IVF), which explains why it is the preferred processing method [7–13].

One of the common causes of infertility in men with normal semen parameters is abnormal sperm DNA. Fertility is not only based on absolute number of spermatozoa but also on the functional capability. A large number of indirect and direct methods are available to measure DNA damage. A positive relationship has been reported between poor semen parameters and DNA damage in spermatozoa indicating inherent problems occurring during spermatogenesis in patients [14]. Earlier studies utilizing TUNEL (terminal deoxytransferase mediated deoxyuridine triphosphate (dUTP) nick end-labeling)-coupled with flow cytometry as well as by comet have shown DNA fragmentation in unselected spermatozoa is related with abnormal sperm morphology [15]. Similar results were reported using the comet test indicating a strong causal link between sperm morphology and DNA damage. The TUNEL assay is increasingly being used in many laboratories [16, 17]. The majority of DNA damage is mainly caused by oxidative stress. Oxidative stress occurs when there is an imbalance between the amounts of reactive oxygen species (ROS) produced and the ability of the antioxidants to remove the excess levels of ROS [18–21]. Oxidative stress can be induced due to infection both viral and bacterial, exposure to xenobiotics, tobacco and alcohol consumption. DNA fragmentation can occur during spermiogenesis. DNA damage can also be caused due to DNA packaging, or due to abortive apoptosis. Post-testicular sperm are more susceptible to DNA damage. The testicular sperm shows the lowest amount of DNA damage; it increases in epididymal and ejaculated sperm [18–21].

Various authors have shown that high DNA fragmentation has an adverse effect on the outcome of assisted reproduction including fertilization rates, embryo cleavage, reduced implantation rate and increased miscarriage rates as well as increased incidence of disease in offspring [14, 22–26]. The DNA damage results as reported by the TUNEL assay are in the range of 15% and 20% [16, 17, 22].

What is not known, however, is whether there is a difference in sperm quality and DNA damage amongst samples processed with various density gradient media. In fact, only one study has examined samples processed with multiple media simultaneously and under identical conditions [27]. Therefore, the aim of this study was to evaluate and compare sperm quality (% motility, total motile sperm (TMS), % recovery and DNA damage) following density gradient sperm preparation with three commonly used, commercially available media: PureCeption, ISolate, and SpermGrad-125.

## Methods

This study was approved by the Institutional Review Board of the Cleveland Clinic. A total of 20 healthy male volunteers of unproven fertility were enrolled in the study (IRB 12-506).

All subjects were young healthy male volunteers aged 21-35 years. They completed a Donor Questionnaire that included multiple yes or no questions such as: undescended testis, mumps, testis infection, testicular injury, varicocele, fever, and sexually transmitted diseases. These donors were selected based on normal semen parameters according to the WHO 2010 criteria for count, motility and morphology. Each donor had at least two of the 3 sperm parameters normal i.e. ejaculate volume, sperm concentration, motility or morphology, to be included in the study. Also all subjects were checked for presence of leukocytospermia i.e. >1×10^6^ white blood cells/mL. Subjects with >1×10^6^ white blood cells/mL were excluded from the study.

Since we were comparing three different gradients, larger volumes were required for the sperm preparation. Additional sample was also required to process sample for measuring DNA damage before and after sperm processing on the three gradients. Therefore, we used individual normal healthy donors without any pooling. Also we chose not to use patient specimen for the comparison of three media to avoid ending up with poor recovery after separation and missing on the important end results, especially in patients with poor semen parameters such as volume, concentration and motility.

### Sample collection

Semen samples were collected from the 20 donors following a period of sexual abstinence of 48 –72 hours. Each donor provided one sample only. Samples were collected in a private collection room at the Andrology laboratory by masturbation into sterile containers. The semen samples were allowed to liquefy completely for 20 minutes at 37°C before further processing. All samples were discarded after the experiment was completed.

### Semen analysis

After liquefaction, a standard semen analysis was performed as per laboratory procedures. The quality of the specimens was assessed by evaluating sperm concentration, motility, morphology, presence of round cells according to WHO criteria [28]. A total amount of 5 μL of the sample was used for manual evaluation of motility using a MicroCell counting chamber (Vitrolife, San Diego, CA) using phase optics at X20 magnification. Equal volume of the same sample was aliquoted and layered on each sperm preparation media and the sperm separation performed under identical experimental conditions.

An aliquot of the sample was saved for measuring DNA damage before sperm processing as described below.

### Sperm DNA damage

**Figure 1:**
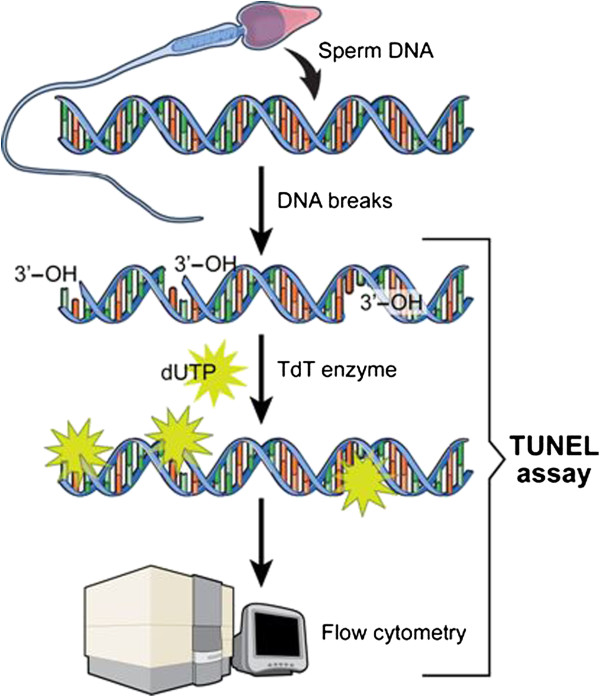
**Schematics of TUNEL assay for measurement of DNA damage in spermatozoa.**

Terminal deoxynucleotidyl transferase-mediated fluorescein-dUTP nick end labeling (TUNEL) using the Apo-Direct™ kit (Pharmingen, San Diego, CA) was used to measure the extent of DNA damage as described earlier [16, 17]. Briefly, 1-2 million spermatozoa were resuspended in 3.7% paraformaldehyde and re-suspended in 70% ice-cold ethanol. Positive and negative kit controls as well as internal controls (specimens from donors and patients with known DNA damage) were included for each run. For the negative control, the TdT enzyme was omitted from the staining step for the positive control, DNA damage was induced by digestion with DNase I. The sample was incubated with 100 μL of DNase I (1 mg/mL) for 1 h at 37°C [29]. The staining solution contained terminal deoxytransferase (TdT) enzyme, TdT reaction buffer, fluorescein isothiocynate tagged deoxyuridine triphosphate nucleotides (FITC-dUTP) and distilled water (Figure 1). All specimens were further washed in rinse buffer to remove the unbound reaction solution, re-suspended in 0.5 mL of propidium iodide/RNase solution, and incubated for 30 minutes in the dark at room temperature followed by flow cytometric analysis.

All fluorescence signals of labeled spermatozoa were analyzed on a flow cytometer (fluorescence activated cell sorting [FACScan] (Becton Dickinson, San Jose, CA). A total of 10,000 spermatozoa were examined for each assay at a flow rate of <100 cells/sec. The excitation wavelength was 488 nm supplied by an argon laser at 15 mW. Green fluorescence (480–530 nm) was measured in the FL-1 channel and red fluorescence (580–630 nm) in the FL-2 channel. Spermatozoa/events obtained in the plots were gated using a forward-angle light scatter (FSC) and a side-angle light scatter (SSC) dot plot to gate out debris, aggregates, and other cells different from spermatozoa. TUNEL-positive spermatozoa in the population were measured after converting the data into a histogram. The percentage of positive cells (TUNEL-positive) were calculated on a 1,023-channel scale using the flow cytometer software FlowJo Mac version 8.2.4 (FlowJo, LLC, Ashland, OR) [16, 17].

### Sperm preparation by density gradient

Three commonly used commercially available density gradients were used. These were PureCeption (Cooper Surgical, Trumbull, CT, United States) with 40% and 80% gradients; ISolate (Irvine Scientific, Santa Ana, CA, United States) with 50% and 90% gradient; and SpermGrad-125 (Vitrolife, San Diego, CA, United States) provided as a single 90% stock solution and then prepared for use as 45% and 90% gradient.

**Figure 2:**
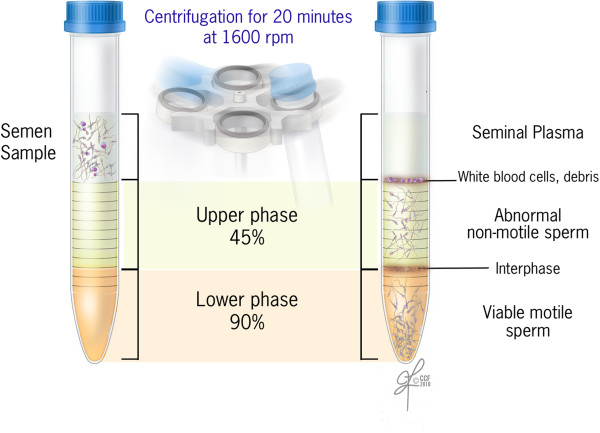
**Schematics of sperm preparation by density gradient separation.**

Components of the density gradient sperm separation procedure included a colloidal suspension of silica particles stabilized with covalently bonded hydrophilic Silane supplied in HEPES. There are two gradients: a lower phase (high density gradient) and an upper phase (low density gradient). Sperm washing medium (HTF; modified HTF with 5.0 mg/mL human albumin) was used to wash and resuspend the final pellet. Briefly, all components of the gradient i.e. upper and lower phase, sperm wash media and semen samples were placed in an incubator at 37°C for 20 minutes for equilibration [5]. Two mL of the lower phase gradient was transferred into a sterile conical–bottom, disposable centrifuge tube. A second 2 mL layer of the upper phase was then gently placed on top of the lower phase using a transfer pipette (Figure 2). A distinct line separating the two layers was observed. Up to 2 mL of liquefied semen was gently placed onto the upper phase. The sample was centrifuged for 20 minutes at 300*g* as described earlier [5]. The supernatant was discarded using a transfer pipette, and a pellet with approximately 0.5 mL was left in the conical-bottom tube. After the first centrifugation, 2 mL of HTF was transferred to resuspend the pellet. The pellet was gently mixed, and the sample was centrifuged for 7 minutes at 300*g*. After removing the supernatant, the final pellet was resuspended in 0.5 mL of HTF. After recording the final volume, the prewash and post-wash total motile sperm (TMS) and percent recovery and percent DNA damage was calculated [5].

Total motile sperm was calculated as follows:


Percent sperm recovery on each sperm preparation media was calculated as follows:


### Statistical analysis

Sperm parameters were compared before and after processing for each media and also after processing amongst the three media groups. All values were reported as mean ± SD. Pairwise comparisons of groups were performed independently after applying Bonferroni correction. Pairwise group comparisons were significant for P < 0.017.

## Results

Semen parameters (mean ± SD) were evaluated for ejaculate volume (3.7 ± 1.5 mL); sperm concentration (X 10^6^/mL) (53.47 ± 32.00); presence of round cells (1.1 × 10^6^/mL); negative for Leukoctyospermia i.e. presence of >10^6^ white blood cells/mL of the seminal ejaculate; sperm morphology (3 ± 2 %) and percent motility (58 ± 9%). Table 1 lists the semen quality parameters percent motility, total motile sperm and recovery rates before and after separation by the three density gradient media.

**Table 1:** **Semen quality parameters before and after separation by three different double density gradients**

Parameter	Pre-wash	Sperm preparation media		
		PureCeption	ISolate	SpermGrad-125
**Motility (%)**	57.8 ± 9.1	62.5 ± 13.2	81.4 ± 6.6^*,**^	85.7 ± 8.0^*,**^
**TMS (X10** ^**6**^ **sperm)**	26.1 ± 16.7	14.2 ± 15.9^*,**^	15.8 ± 18.2^*,**^	10.6 ± 19.7^*^
**Recovery (%)**	-	45.3 ± 18.6^**^	48.9 ± 18.7^**^	30.8 ± 17.2

TMS = total motile sperm; All values are reported as mean ± SD; ^*^,^**^p values were <0.05 was considered significant; ^*^compared with pre-wash; ^**^compared among the gradients.

### Sperm motility

We found significant differences in percent motility between pre-wash and prepared samples for samples prepared by ISolate (P < 0.0001) and SpermGrad-125 (P < 0.0001), while the increase in percent motility for PureCeption was milder and without statistical significance (P = 0.07). After sperm preparation, significantly higher motility was seen for samples prepared by ISolate (P < 0.0001) and SpermGrad-125 (P < 0.0001) compared to those prepared by PureCeption. Percent motility following preparation was slightly higher for SpermGrad-125 than ISolate (P = 0.031).

### Total motile sperm

The number of total motile sperm obtained after separation by PureCeption and ISolate (14.2 ± 15.9 and 15.8 ± 18.2) was higher compared to samples prepared using SpermGrad-125 for TMS (10.6 ± 19.7), though only the difference between ISolate and SpermGrad-125 was statistically significant (P = 0.030).

### Sperm recovery

The percentage recovery of total motile sperm after density gradient centrifugation was significantly higher in sperm prepared by PureCeption and ISolate (45.3% and 48.9%) compared to those separated using SpermGrad-125 (30.8%) (P < 0.01 for each).

### Sperm DNA damage

**Figure 3:**
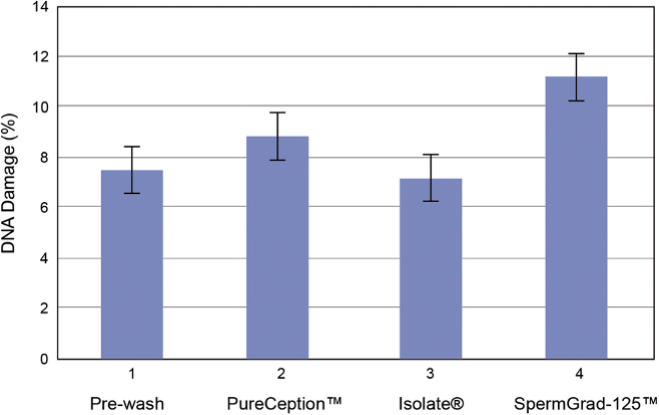
**Schematics of the DNA damage by the TUNEL assay.** (1) Labeling of DNA strand breaks with TdT, which catalyzes the polymerization of labeled nucleotides to free 3′-OH DNA ends in a template-independent manner (TUNEL reaction) and (2) Fluorescein isothiocynate (FITC)-dUTP is incorporated into nucleotide polymers, and it can be directly detected and quantified by flow cytometry.

All unprocessed samples presented low DNA damage (7.5% ± 9.2%). DNA damage was below the established cut-off value of 19% in over 80% (53/60) of the samples before processing. After sperm preparation, DNA fragmentation was comparable in the three gradients (PureCeption™ = 8.8% ± 4.7%, ISolate = 7.2% ± 5.2%, and SpermGrad-125™ = 11.2% ± 7.4%, respectively) (Figure 3).

We also examined the correlation between prewash DNA damage and percent recovery in the post wash sperm. Pureception: pre-wash DNA damage vs. % recovery: r = -0.11, p = 0.65; post-wash DNA damage vs. % recovery: r = 0.01, p = 0.96; Isolate: Pre-wash DNA damage vs. % recovery: r = -0.29, p = 0.24; Post-wash DNA damage vs. recovery: r = -0.34, p = 0.15; SpermGrad: pre-wash DNA damage vs. % recovery: r = -0.29, p = 0.23; Post-wash DNA damage vs. % recovery: r = -0.41, p = 0.09.

## Discussion

The purpose of this study was to determine whether there was a difference in sperm quality after processing with three different density gradient media: PureCeption™, ISolate, and SpermGrad-125. Interestingly, ISolate and PureCeption were associated with the highest TMS and % recovery whereas % motility was higher in Isolate and SpermGrad-125. Pureception is comprised of an 80 percent (vol./vol.) or 40 percent (vol./vol.) sterile colloidal suspension of silica particles which are stabilized with covalently bound hydrophilic silane formulated in HEPES–buffered human tubal fluid (HTF). These new gradients are shown to have better recovery of TMS compared to the earlier formulation of 47% upper and 90% lower densities. In addition, this also contains taurine that promotes capacitation and protects sperm from generation of ROS and prevents against peroxidative damage thereby significantly decreasing the amount of DNA damage. It also contains EDTA, a Ca^2+^ ion chelator which also helps chelate harmful toxic divalent cations as well as reduces the harmful effects of ROS and DNA damage [30]. ISolate is also colloidal suspension of silica particles stabilized with covalently bound hydrophilic silane formulated in HEPES–buffered HTF. It comes as ready to use 50% (upper layer) and 90% (lower layer) concentration, however unlike Pureception, it does not contain any protein supplements. SpermGrad-125 is a stock medium from Vitrolife for preparation of the desired concentration of the upper and lower layers. It is stabilized with bicarbonate and HEPES buffered medium containing silane-coated, colloid silica particles.

Our results showed that all three media improved % motility, TMS, % recovery and DNA damage, which supports previous evidence that density gradient can retrieve high quality motile sperm with little DNA damage [2, 14, 24, 31–33]. However, there was no significant difference in DNA damage in semen specimens prepared from normozoospermic males across the three media. In 2 of our subjects, we observed increased DNA damage (35% and 29%) before sperm preparation; however this was significantly reduced to 20% and 16% respectively after sperm preparation. We anticipate a similar decrease in the extent of DNA damage when using patient specimen for sperm preparation.

Zini et al. [8, 9] compared the sperm preparation by density gradient and swim up methods and found that sperm preparation by density gradient using Percoll resulted in higher percentage of DNA damage compared to those separated by swim-up although both resulted in a superior population of highly motile and normal sperm. These authors attributed this to the production of ROS due to centrifugation and induction of sperm capacitation. Contrary to these findings, another study reported an improvement in DNA integrity following sperm preparation by swim up and density gradient using Pureception [7]. Jayaraman et al. examined 51 subjects with normospermia, oligozoospermia and teratozoospermia and found no difference in the incidence of increased DNA damage in all three groups suggesting comparable results as all resulted in enrichment of sperm with intact chromatin [7].

Zhang et al. [34] reported that sperm preparation by swim up and density gradient using Puresperm resulted in a similar extent of DNA damage, although the stability of sperm separated by density gradient was superior and lasted up to 8 hours compared to those prepared by swim up. They attributed differences in sperm DNA fragmentation dynamics in sperm preparation by different techniques. Similarly, Enciso et al. [13] reported the sperm preparation by density gradient using SpermGrad and by swim up were equally effective in eliminating spermatozoa containing double strand DNA damage and sperm with highly damaged DNA (both single and double strand DNA damage), density gradient was more effective in eliminating single-strand DNA damage compared with swim up technique. Contrary to above reports, Stevanto et al. [35] compared DNA damage before and after sperm preparation using ISolate in 35 patients presenting for ART. These investigators observed an increase in DNA damage in at-least one-third of the subjects, a decrease in another one third and no change in the remaining subjects.

In a study by Chiamchanya et al. [27] three commercially available media were used to compare sperm motility, sperm with normal morphology and DNA damage and protamine deficiency after sperm preparation in 28 infertile patients. The selected sperm preparation media were: PureSperm (Nidacon, Gothenburg, Sweden), Sil-Select Plus (Fertipro, Beernrem, Belgium) and SpermGrad (Vitrolife, Gothenburg, Sweden). The authors reported a significant improvement in sperm motility (both rapid and progressive) and percentage of sperm with normal morphology. PureSperm gave the best results. DNA damage decreased in PureSperm and Sil-Select by 17.9% and 31.3% respectively. Contrary to our studies, they reported a significant increase (56.3%) in DNA damage after separation on SpermGrad. Percentage DNA damage was negatively correlated with percentage of sperm motility, rapid motility and progressive motile sperm concentration. They also examined protamine deficiency and found PureSperm to perform the best. For DNA damage Sil-Select was seen to be the most efficient in reducing DNA damage.

It is especially critical to asses DNA damage before and after sperm preparation individually in subjects presenting for ART as the dynamics of sperm DNA damage may be different depending on the presence of abnormal morphology, sperm concentration and presence of oxidative stress. High levels of DNA damage are positively correlated with lower fertilization and poor implantation rates, miscarriage and recurrent pregnancy loss [10–12, 14, 24, 31, 36, 37].

The current study is significant in that it assessed sperm quality in three sperm preparation media commonly used in infertility clinics under identical conditions. Our results are encouraging that the sperm preparation process irrespective of the type of density gradient used does not induce any significant increase in sperm DNA damage. In a clinical setting, it is extremely important to evaluate DNA damage prior to sperm preparation. Subjects who present with significant amount of DNA damage prior to sperm preparation may exhibit sperm amount of DNA damage after sperm preparation. One limitation of our study was that most of the men (18 of 20) initially presented with low DNA damage (<19%). Examining infertile men who demonstrate DNA damage in their semen samples prior to sperm preparation will be important to rule out DNA damage induced as a result of sperm preparation with these gradients.

## Conclusions

Based on our results we conclude that all three sperm processing media produce samples of good quality and low DNA damage. DNA damage must be evaluated in individual subjects before and after sperm preparation as each subject undergoing ART may present with unique DNA dynamics and the improvement in sperm DNA integrity may be different depending on initial quality of semen such as poor morphology.
